# Experiment Control and Monitoring System for LOG-a-TEC Testbed

**DOI:** 10.3390/s21196422

**Published:** 2021-09-26

**Authors:** Grega Morano, Andrej Hrovat, Matevž Vučnik, Janez Puhan, Gordana Gardašević, Dragan Vasiljević, Tomaž Javornik

**Affiliations:** 1 Jožef Stefan Institute, Jamova 39, SI-1000 Ljubljana, Slovenia; gregaa.morano@gmail.com (G.M.); andrej.hrovat@ijs.si (A.H.); matevz.vucnik@ijs.si (M.V.); 2 Faculty of Electrical Engineering, University of Ljubljana, Tržaška Capital 25, SI-1000 Ljubljana, Slovenia; janez.puhan@fe.uni-lj.si; 3 Faculty of Electrical Engineering, University of Banja Luka, Patre 5, 78000 Banja Luka, Bosnia and Herzegovina; gordana.gardasevic@etf.unibl.org; 4 Mtel a.d. Banja Luka, Vuka Karadžića 2, 78000 Banja Luka, Bosnia and Herzegovina; Dragan.Vasiljevic@mtel.ba

**Keywords:** continuous deployment, testbed, real-time monitoring, Contiki-NG, 6TiSCH, Bluetooth Low Energy

## Abstract

The LOG-a-TEC testbed is a combined outdoor and indoor heterogeneous wireless testbed for experimentation with sensor networks and machine-type communications, which is included within the Fed4FIRE+ federation. It supports continuous deployment principles; however, it is missing an option to monitor and control the experiment in real-time, which is required for experiment execution under comparable conditions. The paper describes the implementation of the experiment control and monitoring system (EC and MS) as the upgrade of the LOG-a-TEC testbed. EC and MS is implemented within existing infrastructure management and built systems as a new service. The EC and MS is accessible as a new tab in sensor management system portal. It supports several commands, including start, stop and restart application, exit the experiment, flash or reset the target device, and displays the real-time status of the experiment application. When nodes apply Contiki-NG as their operating system, the Contiki-NG shell tool is accessible with the help of the newly developed tool, giving further experiment execution control capabilities to the user. By using the ZeroMQ concurrency framework as a message exchange system, information can be asynchronously sent to one or many devices at the same time, providing a real-time data exchange mechanism. The proposed upgrade does not disrupt any continuous deployment functionality and enables remote control and monitoring of the experiment. To evaluate the EC and MS functionality, two experiments were conducted: the first demonstrated the Bluetooth Low Energy (BLE) localization, while the second analysed interference avoidance in the 6TiSCH (IPv6 over the TSCH mode of IEEE 802.15.4e) wireless technology for the industrial Internet of Things (IIoT).

## 1. Introduction

The ever-growing number of Internet of Things (IoT) applications and scenarios using wireless sensor networks (WSN) can be categorized into particular domains, such as smart cities, smart grids, industrial automation, traffic management and logistics, remote monitoring, healthcare and assisted living, agriculture and breeding, public safety, and others. Due to the WSN system complexity and possible interferers, the experimentally-driven research is recognized as a key approach toward the fast and efficient design, development, and deployment of the IoT applications [1]. In this respect, testbeds, which mimic the real but controllable deployment environments, are essential for the experimental research and WSN system development. This was recognized within the European Commission framework Horizon 2020 [2], supporting projects in future internet research and experimentation (FIRE)—for example, the Fed4FIRE+ [3] project, which offers a large federation of next-generation internet (NGI) testbeds consisting of wired and wireless devices.

Deployed WSNs may consist of heterogeneous wireless technologies sharing the same radio resources [4]. In order to mimic the real environment, the wireless testbeds have to support several wireless technologies and enable the execution of various experiments, such as wireless link performance evaluation, custom or standard wireless protocol benchmarking, interference analysis, spectrum sensing, etc. A testbed has to preserve the same test environment for successive experiments and should support automation, real-time monitoring, and control of the experiments by also enabling experiment pausing and stopping. This is of the utmost importance in iterative research processes, such as developing new communication protocols and algorithms. Furthermore, the testbed has to support common tools for the experiment definition, description, control, and execution to enable running the same experiment in various testbeds and radio environments. In addition, the testbed has to be remotely accessible and controlled, in order to provide access to a wide international research community. When a testbed is applied for the evaluation and testing of new devices, protocols, or applications, the testbed’s support of continuous deployment principles is of vital importance [5].

LOG-a-TEC [6], included in a Fed4FIRE+ facilities, combines outdoor and indoor heterogeneous wireless testbeds for experimentation with sensor networks and machine-type communications. It supports continuous deployment principles [5], enabling fully automated experiment execution and results collection, but the interactivity with the nodes has not been fully supported until now. The real-time control and monitoring is very important in the process of designing a software or testing process, which simplifies experiment application debugging and allows for faster comprehension of the results [7]. Moreover, a real-time control of the experiment flow enables the execution of more challenging applications. For instance, when testing the overall performance of the wireless sensor network, it is necessary to establish a fully operational network before executing any tests. However, the moment the fully operational network is established cannot be automatically and precisely detected, unless it is monitored in real-time with the possibility of manually issuing certain commands that influence the network formation, such as setting network coordinator nodes. Thus, to enable the testing and evaluation of all aspects of the wireless sensor networks, apart from automation, it is necessary to provide interactivity in real- or near-real-time. The motivation to upgrade the testbed is two-fold; firstly, provisioning its intuitive usage and better user experience, as well as the inclusion of new functionalities, are all characteristics for wireless environments to run a set of experiments in comparable radio environments. The former includes the design of a new graphical user interface, consisting of the devices monitor, experiment controller with menus, command line input, and textual output. The latter includes additional functionalities: real-time monitoring of the experiment state, option to start or stop the experiment application, reset or restart the nodes in the experiment, monitoring, and controlling the topology of the network under testing.

The contributions of the paper are: (i) a innovative approach, regarding how to complement continuous deployment with near-real-time experiment control and monitoring in distributed wireless testbeds, which does not influence the functionality of continuous deployment and improves the quality of experience for the experimenter, (ii) usage of lightweight ZeroMQ library as an asynchronous messaging framework for control and monitoring the wireless sensor network with minimal system overhead and (iii) novel usage of Contiki-NG shell to control the testbed nodes running the Contiki-NG operating system.

The paper is structured as follows. After the related work, given in the next section, a brief description of the LOG-a-TEC testbed, continuous deployment framework principles, and main shortcomings of the testbed are given in Section 3. Section 4 describes the extension of LOG-a-TEC the testbed with an experiment control and monitor system, which introduces the interactivity in the experimentation. Two examples of the experiments and evaluation of the newly developed tool are illustrated in Section 5. Concluding remarks are drawn in Section 6.

## 2. Related Work

The majority of wireless sensor testbeds include their own version of the monitoring and management system [7]. In order to implement it efficiently, a suitable network protocol for transporting messages between nodes, i.e. the framework, has to be selected. The distributed communication protocols are usually applied [8], which can be classified into two classes, namely client-server protocols and publish-subscribe protocols. Several application layer protocols and their implementations exist [8], among which the most popular are: advanced message queuing protocol (AMQP) [9], message queuing telemetry transport (MQTT) [10], representational state transfer over Hypertext Transfer Protocol (REST/HTTP) [11], and extensible messaging and presence protocol (XMPP) [12]. Some example of different framework implementation are PhyNetLab testbed [13], BOWL testbed [14], NITOS testbed [15] and TARWIS testbed [16].

The PhyNetLab testbed [13] implements a three-tier, real-time distributed architecture, based on REST principles. Tier I consists of the database and other servers, Tier II of gateway nodes, and Tier III of experimental nodes. Some approaches, for example NITOS [15], base their test and management system on a framework solutions, which is a complex integration of several distributed communication protocols. The NITOS applies a control and management framework (OMF), which includes several protocols, i.e., XMPP, REST, and XML-RPC. The architecture is also extended using the WSO2 Enterprise Service Bus to improve the interoperability of different testbed services. NITOS is also part of Fed4Fire testbed federation. In the BOWL testbed [14], the authors presented a management and monitoring system, based on remote method invocation (RMI) with Distributed Ruby. A node controller runs on each node, executing commands initiated by the central node manager. Information and collected states can be extracted from its database and visualized for the user. The authors in [17] proposed an extensible graphical user flow interface for a serviced-defined, network-enabled wireless solution, OpenGUFI, which visualizes the network topology and traffic flows in real-time. The solution is based on open source tools and combines JavaScript Object Notation Remote Procedure Call (JSON-RPC) as a stateless and light-weight RPC protocol, over WebSockets with REST API over HTTP, to effectively manage the distributed system of testbed devices. The framework TARWIS [16] provides control and management functionality, independent from the node type and node operating system. It applies Web Services Description Language (WSDL) as a basis for the communication between components. There are web services and Simple Object Access Protocol (SOAP) libraries for many programming languages. However, in our experience, those can be quite complex and, thus, introduce significant overhead in the system design process.

None of the previously described solution meet the needs for lightweight implementation and do not add perceivable computational and implementation overhead to the existing continuous deployment approach. After careful study of the existing message queue software and frameworks, we selected the open-source ZeroMQ concurrency framework [18]. Its easy-to-use socket, well-documented library provides various flexible and high performance patterns (one-to-one, one-to-many, many-to-many models) and ensures high-speed, asynchronous message exchange [19]. Lightweight and robust design, with ability to run as a part of multi-thread or multi-process application meets the requirements for our implementation of the real-time monitoring tool. Furthermore, some approaches to extract application state information were designed based on TinyOS [7] and do not include support for Contiki-NG and its tool shell, which was our primal target.

## 3. Continuous Deployment Framework for the LOG-a-TEC Testbed

Wireless network development, testing, and deployment calls for testbeds supporting continuous deployment principles, which have to be adjusted to the software and hardware architecture of the particular testbed. In order to better understand the implementation of the experiment control and monitoring system (EC and MS), we briefly review the LOG-a-TEC testbed first and afterwards, the reference continuous deployment architecture is given [5], followed by highlighted deficiencies of the current solution, which motivated this work.

### 3.1. LOG-a-TEC Testbed

**Figure 1:**
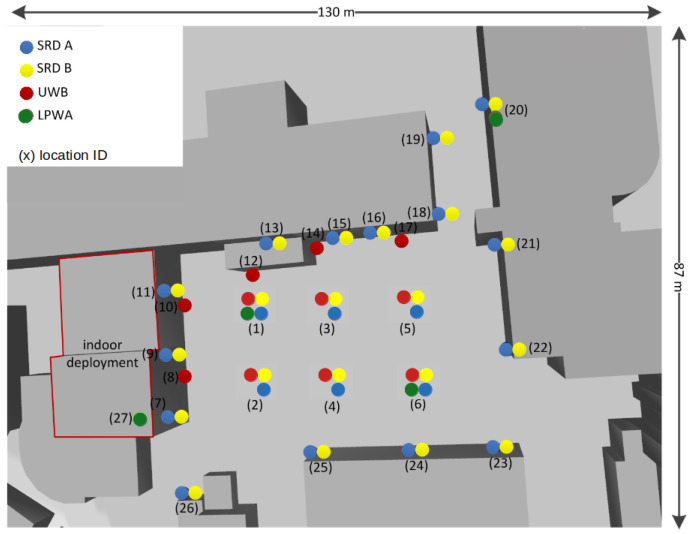
Locations of nodes and available radio technologies in outdoor part of LOG-a-TEC testbed.

LOG-a-TEC, located at Jožef Stefan Institute, Ljubljana, is comprised of several different radio technologies, which, in addition, to traditional IoT and cellular 5G experimentation, supports 5G capillary/MTC experiments, Bluetooth Low Energy (BLE), and WiFi 5 GHz ISM bands. It consists of outdoor and indoor parts. The outdoor part is comprised of 56 physical nodes, which are mounted on the light posts and surrounding building walls, from 2 to 9.3 meters above the ground. The locations of the nodes and available radio technologies in the outdoor part of LOG-a-TEC testbed are depicted in Figure 1. The testbed is also complemented by an additional 31 ultra-wideband (UWB) nodes, deployed indoors and outdoors. The number of locations, their high density, and the possibility that each location can host multiple radio interfaces enables the deployment of very dense and heterogeneous wireless testbed.

Each node in the testbed consists of two functional blocks, namely an infrastructure node and a target node, also termed as a device-under-test (DUT). The infrastructure node is an embedded computer based on the BeagleCore [20] module, running a GNU/Linux operating system named LGTC [21]. It serves as a hosting platform, providing a power supply to the target node, with the ability to manage, control, and program the target node. Infrastructure nodes enable communication with the management server via a wireless backbone. The WiFi module supports dual-band communications on 2.4 GHz and 5 GHz bands. For experimentation purposes, the 2.4 GHz portion of the frequency band is used, while the 5 GHz band is applied for the infrastructure management links [5]. The experimentation node is a custom VESNA (Versatile Platform for Sensor Network Applications) device, based on the ARM Cortex-M3 microcontroller, which can run a dedicated OS (e.g., Contiki-NG and TinyOS) or custom firmware [22]. It can be complemented by application modules with dedicated experimentation transceivers or various sensors. Infrastructure and target nodes are interconnected by a development interface for programming and debugging using JTAG and the application interface for communication using UART. Separating hardware infrastructure, in two functional blocks, enables us to combine a generic infrastructure with various experimentation nodes, in order to support a range of heterogeneous experiments while keeping a unified way of nodes management and reconfiguration [23]. Table 1 shows the number, type, and operating frequencies of currently supported radio interfaces mounted in an indoor and outdoor environment.

### 3.2. The Continuous Deployment Reference Architecture

**Figure 2:**
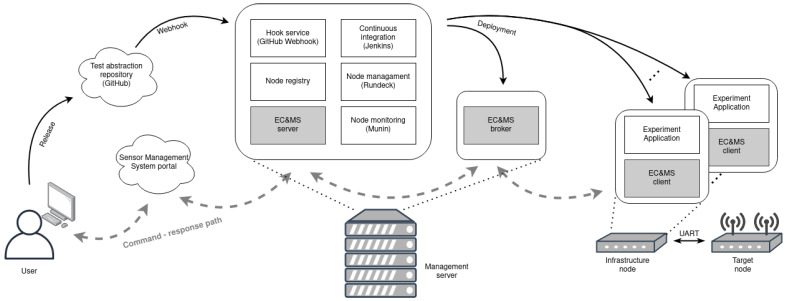
Continuous deployment reference architecture.

The testbed management system architecture [5] consists of the central management and monitoring parts, as well as the part with the experiment deployment and execution system. The central management and monitoring of the testbed, depicted in Figure 2, is achieved by the infrastructure management and build automation system (IMBA). It combines several features, which comprise of testbed device handling and monitoring solutions. It follows the microservice architecture, using self-sufficient systems packaged in Docker [24] containers, and consists of the following services: node registry, node monitoring with MUNIN [25], a hook service with GitHub Webhook [26], a continuous integration service with Jenkins [27], and a node managing toll Rundeck [28]. The access to IMBA services is implemented through a web user interface portal, named the sensor management system (SMS). It is accessible only upon providing valid credentials.

Besides monitoring and managing the nodes, IMBA includes an experiment deployment and execution system, depicted in Figure 2. It is a setup composed of Docker containers and orchestrated with the Ansible engine [29]. Docker containers enable experiment abstraction, so that the required dependencies do not need to be installed on the infrastructure node, and provide an easy way for experiment redistribution [23]. The experiment is deployed when a new release is created in the GitHub repository by triggering GitHub Webhook, depicted in Figure 2. This starts Ansible playbook at IMBA, which contains a description of experiment setup and a list of participating devices. Each infrastructure node downloads the GitHub repository containing a Docker file that includes the actual experiment code. When that Docker image is built, the container is started and the experiment can begin. Although the infrastructure node is devoted to control over the target node, the IMBA generic experiment deployment system supports experimentation with target nodes, as well as with the infrastructure nodes. This feature offers a use of capable Linux-running devices, extending the diversity of the possible experiments with the LOG-a-TEC testbed. The infrastructure node can, furthermore, be extended with any type of USB peripherals. It also supports serial port communication via UART, which is used for the logging of the printf() function-based output made with the target device. At the end of the experiment, all build logs and experiment results are collected using the Ansible engine at IMBA and forwarded to the GitHub, where the user can access the measurements and analyze the received data.

### 3.3. Real-Time Interaction and Monitoring

The described system provides a fully automated experiment execution and result collection, enabling straightforward experimentation and experiment results evaluation. Monitoring the node’s health, used resources, network connection, etc., is implemented by exploiting Muning tool. Rundeck service allows simple node management and provides configuration tools, which keeps the device’s software up-to-date. Continuous integration practices with the Jenkins service offers quick and reliable software development, while continuous deployment offers testing on real hardware. However, with the presented systems, the experimenter has to wait until the end of the experiment, in order to get the information (if the software under testing was working properly). There is no information related to whether the addressed devices received the code and started the experiment or if the results contain the requested measurement.

To enable in-depth testing under comparable conditions, a testbed should provide an environment with a synchronous execution of measurements and ability to start or stop the experiment on multiple nodes at the same time [7]. The current system does not guarantee simultaneous environment preparation, mainly due to the different backbone connection speeds and caching of the Docker container image on each node. Also, the functionalities for restarting the experiment are not accessible. The interactive approach accelerates the debugging of the deployed software and simplifies the development of a new one [7]. While testing new technologies and protocols, there is a need for controlling purposes during experiment execution. For example, when working with Contiki-NG and its protocol stack, usually full operational test network has to be established before executing an experiment. Thus, the information, which and when the chosen devices have joined the test network, is crucial.

With this context in mind, a testbed should provide a comprehensive, post-deployment tool for experiment control and real-time monitoring. Those tools are classified as passive, active, and opportune [30]. Passive tools do not interfere with the test network and cannot interact with the node and its software, while opportune tools rely on the nodes, in order to transmit the measured data in a timely manner. Active tools, on the other hand, require nodes’ interaction and can provide extended communication [30], typically by using serial ports and simple printf() methods. Software of the monitoring tool must be designed in such a way that its impact on the running experiment is minimal. Similar to any other monitoring system, newly developed tools should provide a real-time information exchange mechanism. Seeing that the testbed can be modeled as a distributed system, where each node presents a sub system, a real-time interaction can be achieved with one of the following models for communication in distributed software: message-oriented middleware (MOM), remote procedure calls, remote method invocation, etc. Some of them are already presented in Section 2.

Another aspect to keep in mind, while designing such a tool, is the evaluation of the accumulated data [7]. The use of the graphical user interface is preferable, since the visualization of experiment execution is the most autonomous way of presenting the results.

## 4. LOG-a-TEC Testbed Extension: Experiment Control and Monitor System (EC and MS)

**Figure 3:**
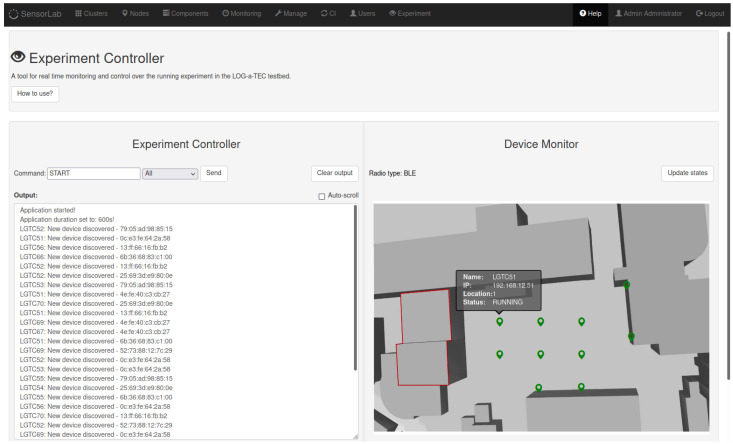
EC and MS graphical user interface during experiment execution.

In order to enable experiment observation and control, we have developed new monitoring systems, called EC and MS. Newly developed functional blocks for continuous deployment frameworks are marked with a light gray color in Figure 2. The user may choose whether to use the monitoring tool in the experiment or not. In the last case, the experiment runs in the same manner as in the previous system. The EC and MS is implemented within the IMBA system as a new service and is accessible to the user via a new tab in the SMS portal [31]. The implemented web graphical user interface is shown in Figure 3. It consists of two functional parts, namely, the experiment controller and the device monitor. The experiment controller is used to issue commands to a targeted device and obtain its responses. Currently, several system commands are supported: start, stop or restart the application, exit the experiment, and reprogram or reset a device. The commands can be sent to a particular device (included in the experiment) or to all devices simultaneously. The response of the devices is given in the experiment controller output window. The device monitor is used to represent the status of devices running the experiment in real-time, as well as their physical position in the testbed. The status of the nodes is represented by different colors of a device symbol. Hoovering with mouse over the symbol displays some additional information about the node, as depicted in Figure 3. Currently, devices in the experiment can be in one of the following states, which can be changed on demand by the user, according to the experiment’s requirements:

- online: device is online and ready for the experiment,
- compiling: infrastructure node is compiling an application for the target node,
- running: experiment is running,
- finished: experiment has come to the end,
- stopped: user successfully stopped the experiment,
- timeout: target node is not responding,
- warning: something went wrong (experiment continues),
- error: an error occurred on the infrastructure node,
- experiment error: an error occurred on the target node.

**Figure 4:**
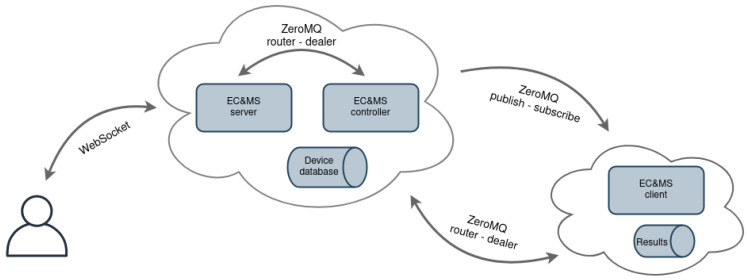
Experiment control and monitoring system reference architecture.

The reference architecture of the EC and MS is shown in Figure 4. It consists of the following entities: EC and MS server, EC and MS controller, and EC and MS client. EC and MS server entity is composed of the Python Flask-SocketIO [32] web application framework, which enables bi-directional communication between the server and users’ browsers, by using the WebSockets protocol [33]. Contrary to pooling methods (AJAX) or server-sent events (SSE), this approach enables sending and receiving commands, without additional delays. The website can be updated by the server whenever any changes emerge in the backend, i.e., in the experiment. The controller part of the EC and MS system is configured and built with each new experiment, as seen in Figure 2. Commands from the user are asynchronously forwarded to the EC and MS controller entity, using the ZeroMQ [18] framework, which provides a high-speed asynchronous message exchange mechanism. It supports multiple messaging protocols, packet buffering, and message patterns with different sockets and can function without a dedicated message broker, which is a typical requirement of other similar MOM software frameworks.

The EC and MS controller processes commands and forwards them to the infrastructure nodes. If a command is intended for one testbed device, the controller entity uses TCP protocol and a ZeroMQ router socket. If a command is targeted for several devices in the testbed at the same time, the controller entity applies multicast protocol and a ZeroMQ publish socket to forward the message. The EC and MS controller also serves as a device database and contains the information regarding all devices and their status; additionally, it enables the user to check the state of the experiment on each device in real-time.

The EC and MS client application runs in its own process on the infrastructure node, ensuring parallel execution with the experiment application. The client receives commands from the EC and MS controller using ZeroMQ subscribe or dealer sockets, processes the commands, and forwards them to the target node via UART connection. The client’s script is also responsible for storing received measurements from target node into a text file, which is at the end of the experiment transmitted to the user for later analysis. The measured data are filtered and forwarded to the EC and MS controller, in order to enable experiment monitoring. Using the EC and MS client, the infrastructure node can now re-compile, flash the target node, and perform a hardware reset on user’s request. When the target node applies Contiki-NG as an operating system, the Contiki-NG shell can be accessed via the experiment monitor [34]. With this feature, it is possible to check the IPv6 address of a device and its neighbours, view the routing tables, issue the command for the network repair, ping neighbour devices, select theDirected Acyclic Graph (DAG) root of the Routing Protocol for Low-Power and Lossy Networks (RPL) network, etc.

## 5. Experiment Control and Monitoring System Functionality Demonstration and Evaluation

The implemented extension of the LOG-a-TEC testbed is demonstrated and evaluated by two experiments. While the first experiment demonstrates the usability of the extension on the infrastructure node by exploiting the BLE interfaces, the second one presents the added value of the upgrade when experimenting with target node. In particular, the solution is evaluated in the terms of additional overhead, delay, experiment repeatability, and achieved goals.

### 5.1. Bluetooth Low Energy Localization

To demonstrate the functionality of EC and MS and its benefits, we developed and deployed an experiment on infrastructure nodes equipped with a WL1837MOD BLE transceiver. Due to its limited range and low power consumption, BLE technology is, in particular, suitable for people’s proximity estimations, especially important in the Covid-19 epidemic. Proximity solutions are based on user terminal tracking, which estimates the distance from the measured, received signal strength to neighbouring terminals. While the estimation of user terminal proximity indoors is, in general, straightforward, due to the high attenuation of room walls, the estimation of the proximity outdoors is a very challenging task. In order to run more complex experiments, we first looked at the estimation of the node location from the BLE beacon received signal strength indicator (RSSI) measurements.

For experimental purposes, we selected 10 nodes, which were asynchronously broadcasting BLE advertising data, while storing all received messages. BLE receivers detected the RSSI of a packet, which were stored along with packet owner’s MAC address. Devices used in this experiment and their approximate positions are depicted in Figure 3.

**Figure 5:**
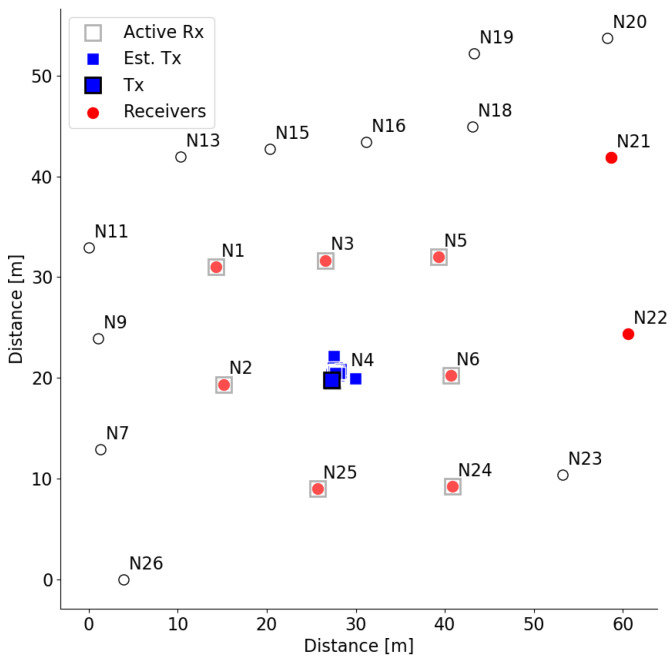
Node location estimated using RSSI BLE measurements.

At the end of the experiment, a custom-designed Python script assembles the measurements from all devices and searches for RSSI measurements advertised from device denoted by N4. Based on the measurements, it calculates the node location and plots the N4 transmitter in the diagram, as depicted in Figure 5. The ground true location of the transmitter is shown as a square with a black edge line, while the estimated transmitter locations are shown as cyan squares without colored edges. The location is calculated using the least-square location algorithm [35]. The distance is estimated from the RSSI, assuming free space path loss propagation with the path loss coefficient equal to 3.8. The estimated location of node N4 is close to its real location. The receiver nodes in this experiment are selected to surround the transmitter node, which leads to good performance.

### 5.2. Interference Avoidance in 6TiSCH IIoT Networks

Functionality of the EC and MS, when experimenting with target nodes, is demonstrated by the experiment for testing the WSN protocols in an industrial environment operating in 2.4 GHz ISM band [36]. In the past, WSNs were foreseen for collecting non-time critical information from the environment, while recently applicable in the industrial Internet of Things (IIoT) domain, the real-time requirements (i.e., low or at least predictable delay and reliability) are of the utmost importance. Due to the harsh industrial radio propagation environment, with strong multi-path and electromagnetic interference [37], the standard IEEE 802.15.4e, specifying the time-slotted channel hopping (TSCH) approach [38,39], has been introduced, in order to support delay-sensitive applications. For the further adaption of IPv6 in industrial standards [40], the 6TiSCH (IPv6 over the TSCH mode of IEEE 802.15.4e) mechanisms are of particular interests.

**Figure 6:**
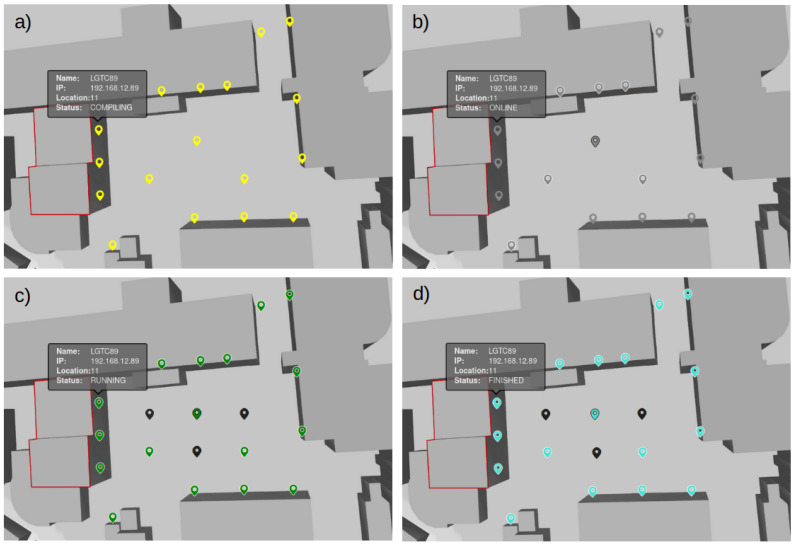
Status of nodes in LOG-a-TEC in experiment run-time: (**a**) code deployment was successful, (**b**) 6TiSCH network joining process, (**c**) nodes are running the experiment (**d**) experiment has come to the end.

Showcase experiment studies the communication capability of 6TiSCH-based WSN in noisy environments. The experiment consists of three parts: (i) observing the performance of the default 6TiSCH network without any interference, (ii) adding the interference and evaluating the behavior of the 6TiSCH network, and (iii) observing the performance of 6TiSCH network with added adaptive channel strategy features under the same interference. In particular we evaluated the approach implemented in Contiki-NG examples, called RSSI upstream-driven adaptive channel selection [41]. In all cases, the root node is configured to periodically send a message to a random device in the network and wait for its response. In order to measure performance, we obtain the packet error rate (PER) at the root node by counting the transmitted and received packets, while storing radio statistics collected at radio driver. In this respect, two networks have been created, namely the 6TiSCH WSN network and interfering network (using three interfering nodes). WSN consists of 17 devices, with an AT86RF231 radio running Contiki-NG OS and a custom-developed application. They are using the 2.4 GHz ISM band, with physical channels in range from 11 to 19. Interfering nodes are equipped with the same AT86RF231 radio, but they are running bare-bone application to generate continuous interference, with a transmit power of 3 dBm on channels 12, 16, and 18. The physical position of the WSNs is depicted in Figure 6, while the interfering nodes are presented in Figure 6c,d with black color.

In both experiments with added interference, we measured approximately the same ratio between the sent and received number of packets as presented in Table 2, thus proving the quality of the 6TiSCH network and its capability of delivering messages even in a noisy environment. However, by looking at the radio driver statistics, we can see much better performance at the 6TiSCH network with the added adaptive channel selection feature. In the default network, the device will try to re-transmit an un-acknowledge message up to 7 times, each time on different channel, until it succeeds. The device in the upgraded network, on the other hand, is avoiding noisy channels before it even tries to transmit on them. The results show that there is 3.5 times less occurrences of re-transmissions, compared to the unmodified version of the 6TiSCH network, which results in more energy efficient communication and smaller packet propagation delay.

### 5.3. Evaluation and Discussion

EC and MS present a tool for the real-time observation of experiment flow and the device states during the experiment execution. If the experiment does not execute according to the expectations, or the device included in the experiment malfunctions (indicated with one of the error/warning states), it can be noticed immediately and the experimenter can take appropriate actions. It is no longer required to wait until the end of the experiment to analyze the execution and operation of the nodes, as when using CD approach without extension. Additionally, EC and MS provides experiment execution and network formation control. Users can start, stop, and restart the experiment with corresponding commands sent to selected nodes. The experimenter can also adapt the network topology to its requirements with Contiki-NG shell commands. For example, in the 6TiSCH experiment, the DAG root of the network is selected with command rpl-set-root, sent to that device. With the command ip-adr, the IP address of the node can be obtained and so forth. New monitoring functionalities enable the user to observe the joining process of all devices and can, therefore, start the measurements only when all devices are within the network, which proved to be crucial in the 6TiSCH experiment. The example of described process is presented in Figure 6a, which shows all infrastructure nodes that received the code of the 6TiSCH experiment and are compiling the application for a target device, indicating that the deployment was successful. In Figure 6b, gray node symbols indicate that the nodes are online and ready for experiment. The root node was selected with command rpl-set-root (marked with a black border around the node symbol) and devices already joined to its network are indicated with a white border. Figure 6c presents the nodes that are running the experiment application; the successful reception of the START command is marked with a green color, and in Figure 6d, the nodes that have finished the experiment are presented with a cyan color.

**Figure 7:**
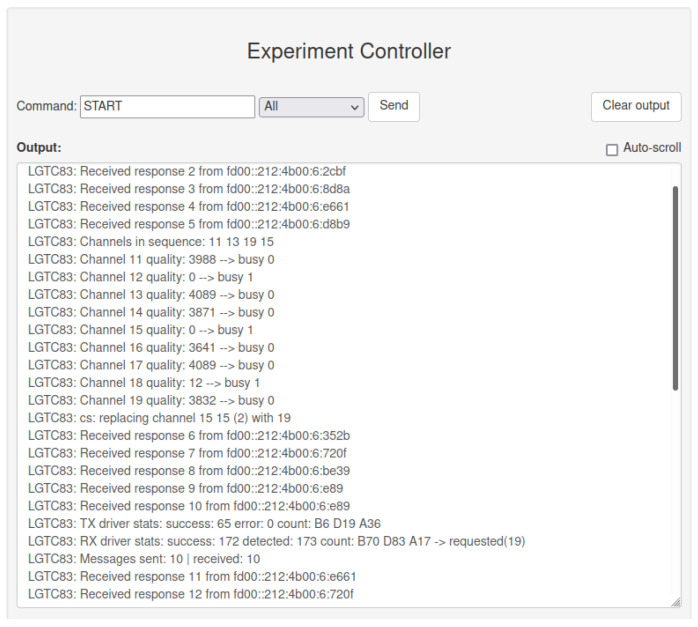
EC and MS output window in experiment run-time for experiment with interference.

During the 6TiSCH experiment, the list of used channels and real-time status of the application in the EC and MS output window, shown in Figure 7, can be observed. The information regarding channel occupancy indicates the proper working of interfering nodes. It additionally helped to analyze the operation of the (upgraded) 6TiSCH protocol and its channel selection in real-time. Driver statistics served to confirm the proper working of the experiment application and its radio drivers. This information was, in previous systems, obtainable only by analyzing the results after the experiment. Moreover, with the help of EC and MS, measured data during the experiment can be easily redirected to the databases, where measurements can be visualized in real-time. For example, this functionality enables the mobile transmitter in the BLE experiment to be tracked in nearly real-time, by extracting the measurements from the database and plotting its position.

With the old deployment system, the nodes start the experiment application automatically, immediately after the process of building the Docker container. Since building process for the individual node can take different amounts of time to complete, depending on the Docker image caching and quality of internet connection on each device, the experiment start can be un-synchronised. With the new monitoring tool, devices receive the application code and build the Docker container (state marked with gray color in the EC and MS monitor window) and wait for incoming START command. Thus, the synchronized start of the experiment execution, which is crucial for experiment repeatability, is now enabled.

Besides proving its essence for software development and protocol testing, the EC and MS monitoring tool further enhanced the usage of CD and improved the user’s experience. The testbed extension enables stopping and restarting the same experiment without triggering the whole experiment deployment process, which enables faster experimentation. On average, it takes approximately 82 s [42] to prepare the experiment environment on the node, while the time needed by the infrastructure node to compile and build the binaries for the target node may take several minutes (the average time to compile the Contiki-NG OS on the infrastructure nodes takes up to 7 min). Consequently, the user must wait around 8 minutes before the target node is ready for experimentation. In the case that the experiment has to be repeated, the additional delay (due to compiling the code and preparing the experimentation environment) is eliminated by the newly introduced solution. In addition, the response of the implemented system was checked by measuring the round-trip time. On average, it takes 648 ms, with a standard deviation of 131 ms, for a command to travel from a user’s browser to the infrastructure device and back and 778 ms, on average, with a standard deviation of 156 ms, for a command to travel to the target device and back. Some deviations are acceptable, since the system is designed in such a way that the experiment has advantages over the user. Because the infrastructure backbone uses Wi-Fi in the 5 GHz range, the additional traffic made by EC and MS does not have an influence on any experimental network of currently supported technologies.

## 6. Conclusions

This paper describes the implementation of the real-time experiment control and monitoring for the LOG-a-TEC testbed and its integration within the continuous deployment process, while keeping the initial continuous deployment functionality unchanged. The newly-developed, real-time experiment monitoring tool supports the observation of the test network formation, issuing the commands to reconfigure the experimentation nodes and the network, as well as for starting/stopping the experiment. It provides an option to check some meaningful variable in real-time and take action upon the received results. The entire measurement procedure can be restarted without issuing a new deployment process. The user can monitor and control the experiment through the web GUI, displaying the status of the particular node and node textual output, defined in the experiment setup in the system output window. Because of its simple design, the user can adapt it to his requirements. Furthermore, the results can be stored in a PostgreSQL database and quarried, which enables the user to build their own near-real-time experiment monitoring application. The functionality of the EC and MS has been demonstrated in two distinct experiments, one using BLE radio technology on infrastructure nodes and the other based on 6TiSCH and Contiki-NG OS (deployed on target nodes). Furthermore, the example experiments demonstrate the importance of iterative research processes, such as developing new communication protocols and algorithms.

## Figures and Tables

**Table 1:** Wireless technologies deployed on testbed [5].

Device	No. Devices Outdoor/Indoor	Freq.	Chip
SRD A	21/0	868 MHz 2400 MHz	AT86RF212 TI CC2500
SRD B	21/0	868 MHz 2400 MHz	TI CC1101 AT86RF231
LPWA	3/1	860 MHz	LoRA SX-1272
UWB	11/20	3500–6500 MHz	DWM 1000
BLE	56/21	2400 MHz	TI WL1837
Management network	56/21	5000 MHz	TI WL1837

**Table 2:** Experiment results.

	PER [%]	No. of Packet Re-Transmissions
6TiSCH	99	103
6TiSCH with interference	95.8	585
Upgraded 6TiSCH with interference	96	166

## Data Availability

Not applicable.

## Abbreviations

The following abbreviations are used in this manuscript:

6TiSCH	IPv6 over the TSCH mode of IEEE 802.15.4e
5G	5th Generation
AJAX	Asynchronous JavaScript and XML
AMQP	Advanced Message Queuing Protocol
API	Application Programming Interface
BLE	Bluetooth Low Energy
CI	Continuous Integration
CD	Continuous Deployment
EC and MS	Experiment Control and Monitoring System
DAG	Directed Acyclic Graph
DUT	Device Under Test
Fed4FIRE+	Federation for Future Internet Research and Experimentation Plus
FIRE	Future Internet Research and Experimentation
GUI	Graphical User Interface
HTTP	HyperText Transfer Protocol
IEEE	Institute of Electrical and Electronics Engineers
IIoT	Industrial Internet of Things
IMBA	infrastructure management and build automation system
IoT	Internet of Things
IP	Internet Protocol
IPv6	Internet Protocol version 6
ISM	Industrial, Scientific and Medical
JSON	JavaScript Object Notation
JTAG	Joint Test Action Group
LGTC	LOG-a-TEC infrastructure node
LPWA	Low Power Wide Area
MAC	Media Access Control
MOM	Message Oriented Middleware
MQ	Message Queuing
MQTT	Message Queuing Telemetry Transport
MTC	Machine Type Communication
NGI	Next-Generation Internet
OS	Operating System
PER	Packet Error Rate
REST	Representational State Transfer
RMI	Remote Method Invocation
RPC	Remote Procedure Call
RPL	Routing Protocol for Low-Power and Lossy Networks
RSSI	Received Signal Strength Indicator
SOAP	Simple Object Access Protocol
SSE	Server Sent Events
SMS	Sensor Management System
SRD A	Short Range Device type A
SRD B	Short Range Device type B
TSCH	Time Slotted Channel Hopping
TCP	Transmission Control Protocol
WiFi	Wireless Fidelity
UWB	Ultra Wide Band
UART	Universal asynchronous receiver-transmitter
USB	Universal Serial Bus
VESNA	VErsatile platform for Sensor Network Applications
WSN	Wireless Sensor Networks
XMPP	eXtensible Messaging and Presence Protocol
